# Paediatric septic thrombophlebitis secondary to an otogenic infection: a single-centre retrospective case series

**DOI:** 10.1007/s00431-026-07040-z

**Published:** 2026-05-15

**Authors:** Arman Nobacht, Yvette G. T. Loeffen, Adriana L. Smit

**Affiliations:** 1 Department of Otorhinolaryngology and Head & Neck Surgery, University Medical Centre Utrecht, Utrecht, the Netherlands; 2 Department of Paediatric Infectious Diseases and Immunology, Wilhelmina Children’s Hospital, Utrecht, the Netherlands; 3 UMC Utrecht Brain Centre, University Medical Centre Utrecht, Utrecht, the Netherlands

**Keywords:** Lemierre’s syndrome, Septic thrombophlebitis, Head-and-neck infections, Otorhinolaryngology, Mastoiditis, Acute otitis media, Anticoagulation

## Abstract

Paediatric septic thrombophlebitis secondary to head and neck infections is a rare but life-threatening disease. Although commonly associated with oropharyngeal infections, otogenic cases have also been described, yet paediatric literature on their disease course and thrombotic distribution remains sparse. To describe the clinical presentation, thrombotic distribution, management, and outcomes of paediatric septic thrombophlebitis secondary to primary otogenic infections. We conducted a retrospective case series of children aged 0–18 years admitted to a tertiary paediatric referral centre between 2016 and 2021 with septic thrombophlebitis following an otogenic infection. Clinical, microbiological, radiological, and treatment data were extracted from records. Ten patients (median age 4.5 years, 70% male) were included. Most cases presented with primary acute otitis media and mastoiditis. *Fusobacterium necrophorum* was detected in 50% of cases. Internal jugular vein thrombosis occurred in 80%, sigmoid sinus thrombosis in 60%, and cavernous sinus thrombosis in 30%. All patients received systemic antibiotics and anticoagulant therapy for medians of 42.5 and 46 days, respectively. Surgery, mainly otogenic interventions, such as mastoidectomy, was performed in 90% of patients. Complications other than thrombosis occurred in 70% of cases and encompassed both intra- and extracranial manifestations. Thrombosis resolved or recanalized in 78% of cases on follow-up imaging.

*Conclusion*: This case series adds to the limited literature on paediatric septic thrombophlebitis secondary to otogenic infections, highlighting a high rate of internal jugular vein thrombosis and a complicated disease course.

**Table:** 

**What is Known:**
• *Paediatric thrombophlebitis secondary to an otogenic infections is a rare but life-threatening disease.*
• *These otogenic presentations can be a diagnostic challenge, while carrying a signifi cant risk of severe intracranial or systemic complications.*
**What is New:**
• *This paediatric case-series describes the complicated disease course of septic thrombophlebitis following primary otogenic infections, demonstrating heterogeneity in clinical presentations and thrombotic distributions.*

## Introduction

Septic thrombophlebitis of the head and neck region is a rare but potentially life-threatening disease in which venous thrombosis is caused by infection and inflammation [1]. Although this disease most commonly affects otherwise healthy adolescents or young adults, paediatric cases also occur, often with atypical disease courses [1]. The typical presentation of septic thrombophlebitis is that of an oropharyngeal infection with subsequent septic spread, resulting in thrombosis in the internal jugular vein, a presentation known as Lemierre’s syndrome [1]. However, septic thrombophlebitis may also develop after otogenic infections, which appear to be relatively more common foci in children than in adults [2, 3]. These otogenic presentations can be a diagnostic challenge, in part because their disease course is still not fully understood, while carrying a significant risk of severe intracranial or systemic complications [4].

The majority of these invasive infections resulting in septic thrombophlebitis are caused by *Fusobacterium necrophorum*, although other pathogens such as Group A Streptococcus have been described [1, 5]. The anatomical site of such infections influences the thrombosis location and the disease course [3]. Although otogenic infections are classically associated with sigmoid sinus thrombosis, internal jugular vein thrombosis has been described as well [3, 6, 7]. In addition to the local thrombosis, the disease course may be complicated by septic embolisms, abscess formation, and invasive spread of infection [8]. In particular, cases of ear infections with *F. necrophorum* are most often characterised by intracranial complications [3].

Literature on the diagnosis and treatment of head-and-neck septic thrombophlebitis remains limited and consists predominantly of case reports, which can be explained by the rarity of the disease [9]. This is particularly true for otogenic paediatric cases, for which evidence regarding the disease course, including thrombotic distribution and propagation, and management remains sparse [10, 11]. Moreover, the high incidence of severe complications highlights our limited understanding of the disease course and how to optimally treat these patients [11]. As such, detailed paediatric case series of otogenic septic thrombophlebitis remain warranted to expand the evidence base and improve clinical awareness to facilitate earlier recognition.

Therefore, this single-centre retrospective case series aimed to describe the clinical presentation, thrombotic distribution, management, and disease course of paediatric septic thrombophlebitis secondary to otogenic infections.

## Method

### Study design, setting, and participants

This single-centre, retrospective case series included children and adolescents aged 0–18 years at onset of symptoms who were diagnosed with a primary ear infection and septic thrombophlebitis and admitted to our tertiary paediatric referral centre, the Wilhelmina Children’s Hospital (WKZ) in the Netherlands. Patients diagnosed with septic thrombophlebitis between January 2016 and December 2021 were included in this study, which reflected the period for case identification and permitted subsequent follow-up assessment. Medical records were assessed in December 2025 for the latest follow-up. The study was performed in accordance with the principles of the Declaration of Helsinki, and the study protocol received a declaration of exemption from the “Medical Research Involving Human Subjects Act” (WMO), meaning that a waiver of ethical approval was granted by the medical-ethical review committee of UMC Utrecht (research number: 19/601, principal investigator: A.S.).

### Data collection

Data from included patients were collected retrospectively by reviewing medical records from the electronic health record (EHR) of children and adolescents diagnosed with septic thrombophlebitis. The registered findings of demographics, medical history, anamnesis, physical examination, therapy, microbiology results, surgery and imaging reports, the medication list, and hospitalization records were used. Both outpatient and inpatient reports were used for this study. The data were pseudonymized and extracted from the medical record by one researcher (AS) and analysed by another researcher (AN). Any discrepancies in the extracted information were resolved by consensus.

### Variables

Demographics included gender and age at onset of symptoms. The prodromal period was defined as the time between the onset of the first symptom(s) of the disease eventually leading to hospital admission. Next, diagnoses at admission to the tertiary paediatric referral centre were listed, and primary infectious foci were anatomically categorized as ear (E), nose (N), and throat (T) infections. The thrombosis location was based on imaging reports. All patients received an initial computed tomography (CT) scan. Follow-up imaging modality (i.e. magnetic resonance imaging (MRI) and/or CT and/or ultrasound) was determined by a multidisciplinary team of clinicians based on the disease course and thrombosis. Thrombosis on follow-up imaging was scored as completely resolved, residual thrombosis with blood flow, and residual thrombosis with complete occlusion. The follow-up period for the thrombosis was defined as the time between the diagnostic scan identifying the thrombosis and the latest follow-up imaging or imaging confirming a completely resolved thrombosis. Pathogen identification was based on blood cultures and local cultures derived from the ear or from a site of septic spread. Hospitalization duration and complications occurring during the disease course were recorded. Finally, the applied treatment (type and duration) was noted and detailed for surgical and non-surgical treatments. Regarding surgical therapy, any sanitizing procedure of the middle ear was classified as a mastoidectomy. For antibiotic treatment, a distinction was made between intravenous, oral, and local antibiotics. Additionally, the type and duration of anticoagulant therapy were recorded.

### Analysis

For analysis of results, descriptive statistics were used. Continuous variables (age, treatment duration, duration of hospitalization, and follow-up time) were visually analysed for normality, using histograms and Q-Q plots. Normally distributed data were presented as mean ± standard deviation (SD) and skewed data were presented as median and interquartile range (IQR). The categorical data of diagnoses and infections at admission, complications during admission, thrombotic complications displayed by site and follow-up outcome, underlying pathogen, and any surgical and non-surgical treatments were presented as frequencies. Analysis was performed with SPSS version 25.0 software (SPSS Inc., Chicago, IL, USA).

## Results

### Studied cohort

Ten patients with a primary otogenic infection and a septic thrombophlebitis were included. The majority were male (70%) and the median age at presentation was 4.5 years (IQR 2.0–6.5), Table 1.

**Table 1:** Demographics, characteristics at presentation and culture outcomes, showing sample sites of positively tested cultures and pathogens

Patient characteristics (*n* = 10)	Number (%)
Demographics	
Age, years	
Median (IQR)	4.5 (2.0–6.5)
Male sex	7 (70%)
Characteristics at presentation	
Prodromal symptoms in days	
Median (IQR)	5.0 (4.0—8.0)
Locus primary infection	
Ear	10 (100%)
Nose	1 (10%)
Throat	0 (0%)
Head&Neck	0 (0%)
Culture	
Sample site	
Ear	5 (50%)
Nose	0 (0%)
Throat	0 (0%)
Local other	1 (10%)
Blood	3 (30%)
Pathogen	
* Fusobacterium necrophorum*	5 (50%)
* Group A streptococcus*	2 (20%)
* Haemophilus influenzae*	1 (10%)
* Streptococcus anginosus and cutibacterium acnes*	1 (10%)
Unknown	1 (10%)

*IQR* interquartile range

### Patient characteristics

The mean duration of the prodromal period was 6.5 ± 3.7 days until hospital admission. The most common prodromal symptoms were fever in 6/10 patients (60%), general illness in 3/10 patients (30%), otalgia in 3/10 patients (30%), and otorrhea in 2/10 patients (20%). Other prodromal symptoms included tiredness, ophthalmalgia, and drowsiness, each observed in one patient (10%).

When patients presented at the tertiary paediatric hospital, the most common symptoms were otorrhea in 10/10 patients (100%), fever in 8/10 patients (80%), and otalgia in 6/10 patients (60%). Other symptoms at presentation were a sore throat in 3/10 cases (30%), otorrhagia in 2/10 cases (20%), and dyspepsia in 1/10 cases (10%). The mean C-reactive protein (CRP) at presentation was 264.5 ± 57.9 mg/L and median leukocyte count was 16.9 × 10^9^/L (IQR, 7.8–23.3).

All patients presented with acute otitis media (100%), with additional mastoiditis in 9 cases (90%). One patient (10%) additionally presented with concurrent sinusitis as a primary infectious focus (Table 1).

*Fusobacterium necrophorum* was the most commonly identified pathogen in patients’ cultures (50%) (Table 1). Cultures also tested positive for *Group A Streptococcus* (20%), *Haemophilus influenzae* (10%), and a combination of *Streptococcus anginosus* and *Cutibacterium acnes* (10%) (Table 1). The pathogen remained unclear for one patient.

### Diagnosis and thrombotic distribution

In all patients a CT scan was performed of the head-and-neck region, which revealed one or multiple venous thromboses, thereby confirming the diagnosis of septic thrombophlebitis. Venous thrombosis was diagnosed with a median of 6 days (IQR, 4.8–9) after onset of symptoms. The thrombotic distribution included the internal jugular vein in 8/10 cases (80%), the sigmoid sinus in 6/10 cases (60%), and the cavernous sinus in 3/10 cases (30%) (Table 2).

**Table 2:** Patient overview of primary source of inflammation, site of septic thrombosis, and complications in the course of the disease

Patient	Primary source of inflammation	Thrombosis location	Complications
1	AOM [R]	Sigmoid sinus to internal jugular vein [R]	Bacterial arthritis, pulmonary abscesses
2	Complicated sinusitis [L + R] and AOM with mastoiditis [L]	Sigmoid sinus to internal jugular vein [L]	Retropharyngeal and retro-orbital abscess, septic pulmonary embolisms, orbital cellulitis [L]
3	AOM with mastoiditis [L]	Cavernous sinus [L + R]	Osteomyelitis, os petrosum abscess
4	AOM [L]	Internal jugular vein and partial cavernous sinus [L]	Bacterial meningitis, osteomyelitis, cavernous sinus abscess
5	AOM with mastoiditis [L]	Sigmoid sinus to internal jugular vein [L]	Bacterial meningitis
6	AOM [L + R]	Cavernous sinus [L]	Abscesses around left internal carotid artery
7	AOM [L + R] with mastoiditis [L]	Sigmoid sinus to internal jugular vein [L]	-
8	AOM with mastoiditis [L]	Sigmoid sinus to internal jugular vein [L]	-
9	AOM with mastoiditis [L]	Internal jugular vein [L]	Aseptic meningitis
10	AOM with mastoiditis [R]	Sigmoid sinus and transverse sinus to internal jugular vein [R]	-

*AOM* acute otitis media

### Treatment

#### Antibiotics

Antibiotic treatment involved multiple agents via different routes, as shown in Table 3. Prior to admission to the study centre, oral antibiotics were prescribed before intravenous antibiotics in 3/10 cases (30%). Patients received intravenous antibiotics for a total duration of 21.0 days (median; IQR, 18.5–31.8). Empirical intravenous antibiotics consisted of ceftriaxone plus metronidazole in 9/10 patients (90%) and flucloxacillin plus gentamicin in one patient (10%). Afterwards, other prescribed intravenous antibiotics were amoxicillin in 5/10 cases (50%), benzylpenicillin in 2/10 cases (20%), flucloxacillin in 1/10 cases (10%), and amoxicillin and clavulanic acid in 1/10 cases (10%). Oral antibiotics were prescribed for a median of 21.5 days (IQR, 0.0–38.3), comprising amoxicillin and clavulanic acid in 4/10 cases (40%) and metronidazole in 2/10 cases (20%). Local antibiotics (eardrops) consisted of framycetin-gramicidin-dexamethasone in 8/10 cases (80%), ofloxacin in 4/10 cases (40%), and hydrocortisone-bacitracin-colistin in 4/10 cases (40%) and were given for a median duration of 26.5 days (IQR, 11.8–44.5). After discharge, antibiotics were continued intravenously in 7/10 (70%) patients (median 5.5 days; IQR, 0.0–15.8), orally in 5/10 (50%) patients (median 10.0 days; IQR, 0.0–24.0). Antibiotics were locally continued in 9/10 (90%) patients (median 7 days; IQR, 4.0–23.0) and discontinued in one patient. Total duration of systemic antibiotic treatment was 42.5 days (median; IQR, 18.5–50.3).

**Table 3:** Treatment overview per patient of case conclusion, performed surgeries, prescribed intravenous, oral and local antibiotics with total duration and prescribed anticoagulants with total duration

Patient	Conclusion	Surgery	Antibiotics i.v. (days)	Antibiotics oral (days)	Antibiotics local (days)	Anticoagulants (days)
1	AOM with mastoiditis complicated by bacterial oligoartritis, pulmonary abscesses and SS to iJV thrombosis	Drainage arthritis hip + Mastoidectomy + tymp (R)	Flu, Gen, Ben (17)	-	FGD, Ofl (29)	Dal, Fen (204)
2	AOM with mastoiditis and sinusitis and complicated by orbital cellulitis, a retropharyngeal and retroorbital abscess, septic pulmonary embolisms and SS to iJV thrombosis	FESS + Mastoidectomy + drainage abscesses + tymp (L + R)	Cef, Met, Am, Cla, Cli (27)	Am, Cla (38)	FGD (49)	Dal, Fen (43)
3	AOM with mastoiditis complicated by osteomyelitis, cavernous sinus thrombosis, os petrosum abscess	Mastoidectomy + drainage abscess + tymp (L + R)	Cef, Met, Gen (21)	Am, Cla (20)	HBC (43)	Dal (49)
4	AOM with mastoiditis complicated by bacterial meningitis, osteomyelitis, cavernous sinus abscess and iJV and partial CS thrombosis	Mastoidectomy + tymp (L)	Cef, Met, Am, Cla, Flu (50)	-	FGD, Ofl, HBC (98)	Dal (40)
5	AOM with mastoiditis complicated by bacterial meningitis and SS to iJV thrombosis	Mastoidectomy Mastoidectomy + tymp (L)	Cef, Met (19)	-	HBC, Ofl (24)	Dal (17)
6	AOM complicated by abscesses around internal carotid artery and CS thrombosis	-	Cef, Met (6)	Am (2)	HBC, FGD (7)	Dal (25)
7	AOM with mastoiditis complicated by SS to iJV thrombosis	Mastoidectomy + tymp (L + R)	Cef, Met, Am, Cla, (27)	Am, Cla (30)	FGD (30)	Dal (21)
8	AOM complicated by bilateral mastoiditis and SS to iJV thrombosis	Mastoidectomy + tymp (L + R)	Cef, Met, Am, Cla, (46)	Met (39)	FGD, Ofl (20)	Dal, Fen (120)
9	AOM with mastoiditis complicated by aseptic meningitis and iJV thrombosis	Mastoidectomy + tymp (L)	Cef, Met, Am, Ben (21)	Am, Cla Met (41)	FGD (13)	Dal (101)
10	AOM with mastoiditis complicated by SS and TS to iJV thrombosis	Mastoidectomy + tymp (L + R)	Cef, Met (21)	Am, Cla (23)	FGD (8)	Dal (50)

*AOM* acute otitis media, *CS* cavernous sinus, *iJV* internal jugular vein, *SS* sigmoid sinus,  *tymp* surgery: tympanostomy, *Am* antibiotics: amoxicillin, *Ben* benzylpenicillin, *Cef* ceftriaxone, *Cla* clavulanic acid, *Cli* clindamycine; *Flu* flucloxacillin, *FGD* framycetin-gramicidin-dexamethasone, *Gen* gentamicin, *HBC* hydrocortisone-bacitracin-colistin, *Met* metronidazole, *Ofl* ofloxacin,  *Dal* anticoagulation: dalteparine, *Fen* fenprocoumon

#### Surgery

Surgery was performed in 9/10 (90%) patients, which included a mastoidectomy in all nine patients with clinical mastoiditis (Table 3). A tympanostomy was performed in 9/10 (90%) of cases; five of these were bilateral (Table 3). In two patients, drainage of an abscess (located retropharyngeal and retro-orbital, and os petrosum abscess, respectively) was performed. The patient with sinusitis required functional endoscopic sinus surgery (FESS). Three patients (30%) required a second-look surgery for tympanostomy tube (re)placement because of extrusion or blockage during the course of the disease.

#### Anticoagulation

All patients received anticoagulant therapy after diagnosis of thrombosis. Median duration of anticoagulant therapy was 46.0 days (IQR, 24.0–105.8). Initial treatment consisted of low molecular weight heparin (dalteparin) for a median of 32.5 days (IQR, 21.0–52.0). Three patients (30%) received subsequent anticoagulant therapy with a vitamin K antagonist (phenprocoumon), Table 3.

### Complications

In 7/10 patients (70%), other septic-embolic complications were diagnosed as shown in Table 2. This included three cases (30%) of meningitis. Osteomyelitis was diagnosed in one patient with mastoiditis and os petrosum abscess (10%). One patient with internal jugular vein thrombosis developed pulmonary embolisms and a pulmonary abscess. Other complications were bacterial arthritis, a cavernous sinus abscess, abscesses around the left internal carotid artery, and retropharyngeal and retro-orbital abscesses, each observed in one patient (10%).

### Follow-up

Patients were hospitalized for a mean of 18.1 ± 5.9 days. One patient, initially admitted with mastoiditis and treated with mastoidectomy and tympanostomy tube placement, required a 4-day readmission due to relapsing acute otitis media and tube obstruction, necessitating tube replacement. Follow-up imaging of the thrombosis was available in 9 out of 10 cases, with the latest imaging after a mean of 71.4 ± 26.6 days. Thrombosis completely resolved in 4/9 patients (44%), showed residual flow in 3/9 (33%), and completely occluded the vein(s) in 2/9 (22%). All patients recovered fully without long-term sequelae.

## Discussion

In this single-centre retrospective case series, we describe the complex disease course of septic thrombophlebitis in ten children with an otogenic source of infection. In this cohort, (1) internal jugular vein thrombosis was the predominant thrombotic manifestation; (2) intracranial complications were common; and (3) management was multimodal, combining surgery, antibiotics, and anticoagulation.

### Primary infectious foci and pathogenesis

In our paediatric cohort, otogenic infections, particularly acute otitis media and mastoiditis, represented the predominant primary foci. This reflects our institution’s role as a tertiary referral centre for paediatric otology. One patient additionally presented with concurrent sinusitis as a primary infectious focus, illustrating that the underlying primary infections can be heterogeneous. The microbiological findings were in line with previous literature, identifying *Fusobacterium necrophorum* as the most common pathogen, consistent with its well-recognised virulence and role in endothelial injury and thrombus formation [3]. Yet, the detection of *Group A Streptococcus* and *Haemophilus influenzae* highlights the polymicrobial nature of this disease. These findings support broad initial microbiological evaluation, including anaerobic testing, in all suspected cases.

### Diagnosis and imaging

In this cohort, the interval from prodromal symptom onset to diagnosis of septic thrombophlebitis was approximately 2 weeks, aligning with previously described 7 to 14 days from initial symptoms to systemic complications [8]. This delay may reflect the complexity of this condition, particularly in paediatric populations where prodromal symptoms often overlap with those of uncomplicated ENT infections. Moreover, classical diagnostic indicators of internal jugular vein thrombosis, such as neck swelling, were often absent. This was also reported in nearly half of the 109 cases reviewed by Chirinos [12]. Furthermore, as the progression from otogenic infection to septic thrombophlebitis likely occurs over several days, worsening or evolving symptoms should prompt clinicians to consider the diagnosis and pursue appropriate imaging.

While all patients underwent CT imaging for diagnosis, MRI is considered the gold standard for diagnosing cerebral venous thrombosis [13]. The reliance on CT may have affected diagnostic accuracy, particularly for smaller thrombi or early-stage thrombosis. Follow-up imaging modality varied by thrombosis location, with ultrasound used for isolated internal jugular vein thrombosis and MRI for intracranial thrombosis. This may have affected the accuracy of thrombosis assessment.

### Distribution of thrombosis

Internal jugular vein thrombosis was notably frequent, occurring in 8 out of 10 cases and exceeding the incidence of intracranial venous sinus thrombosis, including the sigmoid and cavernous sinuses. This contrasts with the traditional description of otogenic infections as primarily associated with sigmoid or transverse sinus thrombosis [3]. It is well-established that otogenic infections typically cause sigmoid or transverse sinus thrombosis that can propagate anteriorly into the internal jugular vein [7]. However, the relatively lower incidence of sigmoid sinus thrombosis in this case series suggests that internal jugular vein thrombosis may not solely depend on such propagation [6]. Notably, our retrospective study design does not allow us to determine whether the observed internal jugular vein thrombosis represents: (1) retrograde extension from primary sigmoid sinus thrombosis, (2) independent thrombosis due to local inflammation, or (3) earlier detection with modern imaging protocols.

### Treatment

The treatment regimens used in this case series reflect a multidisciplinary yet non-standardised approach to management. However, creating standardised protocols remains challenging due to the rarity of the disease and the lack of high-level evidence.

All patients received systemic antibiotics, most commonly ceftriaxone plus metronidazole, for a median duration of 43 days. This is broadly consistent with previous literature, describing durations of 4 to 6 weeks for septic thrombophlebitis and Lemierre’s syndrome [14]. Moreover, this represents an established strategy to cover both aerobic and anaerobic pathogens, including *Fusobacterium necrophorum* [11]. However, such recommendations are based mainly on expert opinion and retrospective data rather than controlled trials.

Surgery was required to eradicate the infectious foci in all but one patient, most commonly involving mastoidectomy [14]. The need for second-look procedures likely reflects the dynamic clinical course of severe otogenic infections rather than indicating treatment failure alone, highlighting the importance of close clinical monitoring to prevent recurrence or residual disease despite initial management.

The role of anticoagulation in septic thrombophlebitis remains highly controversial, with no consensus on its necessity [1, 15, 16]. Although anticoagulation is frequently used in septic thrombophlebitis, its therapeutic benefit remains uncertain [17, 18]. The American Heart Association/American Stroke Association states that antibiotic therapy and surgical intervention are the mainstays of treatment for septic cerebral venous sinus thrombosis, while the role of anticoagulation remains controversial [19]. In our cohort, anticoagulation was initiated following radiological confirmation of thrombosis in all patients, though optimal timing relative to surgical intervention remains undefined. Notably, recanalization rates of up to 80% have been reported in patients with antibiotics and surgery alone [4, 18, 19]. Thrombosis resolved or recanalized in 78% of our cohort, highlighting the difficulty in attributing these outcomes to anticoagulation specifically. Nevertheless, in the absence of contraindications, observational studies suggest that anticoagulation may improve thrombotic outcomes without increasing major bleeding events [2]. Regarding safety, no major bleeding complications attributable to anticoagulation were documented in our cohort. Emerging evidence also supports the use of direct oral anticoagulants as an alternative in paediatric patients [20–22].

### Complications

Intracranial complications, including meningitis and intracranial abscess formation, occurred in 60% of cases, reflecting the anatomical proximity of the infectious foci to venous structures and the brain [3]. Moreover, the low incidence of pulmonary embolism (10%) is consistent with the typical disease course of septic thrombophlebitis following an otogenic infection [3]. Further complications, including osteomyelitis and retro-orbital abscesses, highlight the contiguous spread of infection and illustrate the complex disease course of otogenic septic thrombophlebitis.

## Limitations

This case series has several limitations. First, the single-centre setting and small sample size limit generalisability and may not be representative of other centres or populations. For example, percentages derived from such small numbers can be misleading. Second, the retrospective design introduces selection bias and limits our ability to assess causality, particularly regarding treatment decisions such as anticoagulation. Third, the absence of a non-anticoagulation control group prevents assessment of whether anticoagulation improved outcomes beyond antibiotics and surgery alone. Fourth, follow-up imaging was inconsistent in timing and modality, limiting conclusions about recanalization rates. Finally, the absence of long-term functional outcomes restricts the conclusions that can be drawn about the broader impact of this condition on paediatric patients. Nevertheless, the rarity of this condition makes conducting large prospective studies or clinical trials challenging. By describing multiple cases in detail, this study may contribute to future meta-analyses to strengthen the evidence base for the management of paediatric septic thrombophlebitis.

## Conclusion

In this retrospective paediatric case series, we describe the complicated disease course of septic thrombophlebitis following primary otogenic infections. Notably, we report heterogeneous clinical presentations and thrombotic distributions, including a high frequency of internal jugular vein thrombosis, thus adding to the limited literature on paediatric septic thrombophlebitis secondary to otogenic infections. Our findings underscore the importance of a multidisciplinary approach that includes antibiotic therapy, surgical intervention, and judicious use of anticoagulation. Moreover, continued clinical vigilance is essential in children with persistent or worsening otogenic infections, including acute otitis media, to enable early recognition of septic thrombophlebitis.

## Data Availability

No datasets were generated or analysed during the current study.
